# Allogeneic Hematopoietic Stem Cell Transplantation in Immunodeficiency—Centromeric Instability—Facial Dysmorphism (ICF) Syndrome: an EBMT/ESID Inborn Errors Working Party Study

**DOI:** 10.1007/s10875-024-01786-7

**Published:** 2024-08-21

**Authors:** Dagmar Berghuis, Lubna S. Mehyar, Rolla Abu-Arja, Michael H. Albert, Jessie L. Barnum, Horst von Bernuth, Reem Elfeky, Philippe Lewalle, Alexandra Laberko, Sujal Ghosh, Mary A. Slatter, Corry M. R. Weemaes, Akif Yesilipek, Tiarlan Sirait, Bénédicte Neven, Andrew R. Gennery, Arjan C. Lankester, on behalf of the Inborn Errors Working Party (IEWP) of the European Society for Immunodeficiencies (ESID), the European Society for Blood and Marrow Transplantation (EBMT) and the European Reference Network that aims at improving the care of patients with rare immunological disorders (ERN-RITA)

**Affiliations:** 1 Willem-Alexander Children’s Hospital, Department of Pediatrics, Division of Pediatric Immunology, Hematology and Stem Cell Transplantation, Leiden University Medical Center, Leiden, The Netherlands; 2 Division of Pediatric Hematology, Oncology, Blood and Marrow Transplant, Dayton Children’s Hospital/Department of Pediatrics, Boonshoft School of Medicine, Wright State University, Dayton, OH USA; 3 The Blood and Marrow Transplant Program, Nationwide Children’s Hospital, Colombia, OH USA; 4 Department of Pediatrics, Dr. Von Hauner Children’s Hospital, University Hospital, LMU, Munich, Germany; 5 Division of Blood and Marrow Transplantation and Cellular Therapies, University of Pittsburgh Medical Center (UPMC) and Children’s Hospital of Pittsburgh, Pittsburgh, PA USA; 6 Charité-Universitätsmedizin Berlin, Corporate Member of Freie Universität Berlin, Humboldt-Universität zu Berlin, Department of Pediatric Respiratory Medicine, Immunology and Critical Care Medicine, University Hospital Center, Berlin, Germany; 7 Department of Immunology, Labor Berlin GmbH, Berlin, Germany; 8 Charité-Universitätsmedizin Berlin, Corporate Member of Freie Universität Berlin, Humboldt-Universität zu Berlin, and Berlin Institute of Health (BIH), Berlin-Brandenburg Center for Regenerative Therapies (BCRT), Berlin, Germany; 9 Great Ormond Street (GOS) Hospital for Children National Health Service (NHS) Foundation Trust, University College London Great Ormond Street (GOS) Institute of Child Health, and National Institute for Health and Care Research (NIHR), Great Ormond Street Hospital (GOSH), Biomedical Research Centre (BRC), London, UK; 10 Hematology Department, Hopital Universitaire de Bruxelles (HUB)-Institut Jules Bordet, Brussels, Belgium; 11 Department of Hematopoietic Stem Cell Transplantation, Oncology and Immunology, Dmitry Rogachev National Medical Research Center of Pediatric Hematology, Moscow, Russia; 12 Department of Pediatric Oncology, Hematology and Clinical Immunology, Medical Faculty, Center of Child and Adolescent Health, Heinrich-Heine-University, Duesseldorf, Germany; 13 Children’s Haemopoietic Stem Cell Transplant Unit, Great North Children’s Hospital, Newcastle-upon-Tyne Hospital NHS Foundation Trust, Newcastle Upon Tyne, UK; 14 Department of Pediatrics, Radboud University Medical Centre Nijmegen, Nijmegen, the Netherlands; 15 Department of Pediatric Hematology and Pediatric Stem Cell Transplantation Unit, Medicalpark Antalya Hospital, Antalya, Turkey; 16 EBMT Leiden Study Unit, Leiden, The Netherlands; 17 Immuno-hematology and rheumatology Unit, Necker Children Hospital, Imagine Institute, UMR 1163, University of Paris Cite, Paris, France

**Keywords:** Immunodeficiency–centromeric instability–facial dysmorphism (ICF) syndrome, Hematopoietic stem cell transplantation, Combined immunodeficiency, Pre-emptive

## Abstract

Immunodeficiency–Centromeric instability–Facial dysmorphism (ICF) syndrome is an inborn error of immunity characterized by progressive immune dysfunction and multi-organ disease usually treated with antimicrobial prophylaxis and immunoglobulin substitution. Allogeneic hematopoietic stem cell transplantation (HSCT) is the only curative treatment, but data on outcome are scarce. We provide a detailed description of disease characteristics and HSCT outcome in an international cohort of ICF syndrome patients. Eighteen patients (including all four genotypes) were enrolled. Main HSCT indications were infections (83%), enteropathy/failure to thrive (56%), immune dysregulation (22%) and myelodysplasia/haematological malignancy (17%). Two patients underwent pre-emptive HSCT after early diagnosis. Patients were transplanted between 2003–2021, at median age 4.3 years (range 0.5–19), after myeloablative or reduced-intensity conditioning, from matched sibling or matched family donors, matched unrelated or mismatched donors in 39%, 50% and 12% of cases respectively. Overall survival was 83% (all deaths occurred within the first 5 months post-HSCT; mean follow-up 54 months (range 1–185)). Acute GvHD occurred in 35% of patients, severe (grade III) in two (12%), while none developed chronic GvHD. At latest follow-up (median 2.2 years (range 0.1–14)), complete donor chimerism was achieved in 15/17 surviving patients. All survivors demonstrated normalized T and B cell numbers. Immunoglobulin substitution independence was achieved in all but two patients. All survivors recovered from pre-transplant infections, enteropathy/failure to thrive and immune dysregulation. All three patients transplanted at young age (≤ 3 years), after early diagnosis, survived. The favourable clinical and immunological HSCT outcome in this cohort of patients supports the timely use of this curative treatment in ICF syndrome.

## Introduction

Immunodeficiency – centromeric instability – facial dysmorphism (ICF) syndrome is a rare and heterogeneous autosomal recessive inborn error of immunity. Pathogenic variants in four genes have been identified to cause ICF syndrome: *DNA methyltransferase 3B* gene (*DNMT3B;* ICF1), *Zinc-finger and BTB domain-containing 24* gene (*ZBTB24*; ICF2), *cell division cycle associated 7* gene (*CDCA7*; ICF3) and *helicase lymphoid specific* gene (*HELLS*; ICF4). The molecular hallmark of ICF syndrome is DNA hypomethylation of pericentromeric satellite repeats, resulting in characteristic chromosomal aberrations of predominantly chromosomes 1, 9 and 16 [1–3].

The immunodeficiency in ICF syndrome is characterized by recurrent infections of predominantly the airways and gastrointestinal tract, caused by a variety of pathogens including bacteria, viruses and fungi. The immunological hallmark of ICF syndrome is a humoral immunodeficiency: patients almost invariably present with hypo- or agammaglobulinemia and inadequate antibody responses to vaccines, in the presence of normal numbers of circulating total B cells. Reduced numbers of switched memory B cells, most likely caused by a defect in the terminal stages of B cell differentiation, seem responsible for this humoral immunodeficiency [2, 4, 5]. In addition to this humoral immunodeficiency, the co-existence of an intrinsic T cell defect has been presumed in several patients with ICF syndrome presenting with severe viral and/or opportunistic infections (including herpes viruses, adenovirus, Pneumocystis jirovecii and Candida), manifestations of immune dysregulation (including hepatitis, cytopenia, enteropathy, often without detectable auto-antibodies) or (haematological) malignancy. Indeed, immunological profiling of these patients has demonstrated impaired T cell function including decreased T cell numbers and/or defective lymphocyte mitogen responses that may progress over time. These findings and the reported impact of antimicrobial prophylaxis and immune suppression in these patients support the existence of a combined rather than humoral immunodeficiency in these patients with ICF syndrome [1, 2, 6–12]. Recent studies, as reviewed by Unoki et al. [13], have provided clues to understanding the pathophysiology underlying the combined immunodeficiency in ICF syndrome, including pivotal roles for ICF-related proteins in non-homologous end joining during double-strand DNA break repair, immunoglobulin class switch recombination and dysregulation of immunoglobulin signaling. However, further studies are needed to unveil the exact mechanisms underlying combined immunodeficiency in ICF syndrome.

IgG replacement therapy and antimicrobial prophylaxis/therapy represent the cornerstone for treatment of patients with humoral immunodeficiencies, and these therapies have been applied in most patients with ICF syndrome as well. However, a substantial number of patients with ICF syndrome demonstrate severe or progressive disease manifestations despite these supportive therapies. Overall survival rates of 60–84% are reported in several (older) cohorts of predominantly non-HSCT-treated ICF patients [1, 9, 14]. While ICF syndrome represents a multisystem disease, clinical deterioration or death usually result from severe infections, immune dysregulation or (haematological) malignancy. To date, several case reports have been published on allogeneic haematopoietic stem cell transplantation (HSCT) as curative therapeutic option for severely affected ICF syndrome patients [2, 8, 11, 15–20]. However, a comprehensive overview on clinical, immunological and HSCT characteristics of transplanted patients is currently lacking.

In this retrospective study, we provide a detailed description of the disease characteristics and HSCT outcomes of an international cohort of 18 patients including all four genotypes of ICF syndrome.

## Methods

We performed a retrospective analysis of HSCT-treated patients with ICF syndrome. Centers with patients were identified through the Inborn Errors Working Party of the European Society for Immunodeficiencies (ESID) and European Society for Blood and Marrow Transplantation (EBMT), the EBMT registry (study number 8427015), published case reports and communication with expert clinicians working in the field. Study approval was granted by the scientific committee of the Inborn Errors Working Party (IEWP) of the EBMT and the board of directors of the Medical Ethics Committee Leiden The Hague Delft (August 3rd, 2020; reference number G20.095). A specific questionnaire for data collection and analysis was distributed among the participating centers. Data of all patients registered at the EBMT office were in compliance with the General Data Protection Regulation (GDPR 2016/679). Data for patients previously enrolled in non-EBMT member centers were retrieved and shared in irreversibly de-identified form after informed consent from patients and/or families was obtained in accordance with the Declaration of Helsinki. Data collected included clinical, genetic and immunological characteristics before transplant; HSCT characteristics; outcome regarding engraftment, chimerism, immunological reconstitution and clinical status after transplant.

Analysis was performed using data collected for 18 patients from 12 centers worldwide, transplanted between 2003 and 2021. Diagnosis of ICF syndrome, including genetic subtype, was made based on molecular genetics or clinical characteristics in two cases (ESID Registry criteria [21]). Limited data from nine patients have been previously published: P1[15], P4[15], P15[15], P5[17], P2[20], P7[16], P9[19], P13 [11] and P14[11]. In this study, detailed clinical, immunological, HSCT and long term outcome data were collected for all patients. Analyses were performed using SPSS statistical software (IBM SPSS Statistics, version 29).

The conditioning regimen was categorized as myeloablative and reduced intensity, in accordance with the IEWP guidelines [22]. Based on high resolution HLA typing, donors were grouped into four categories: matched sibling donor and matched family donor (10/10 identical relatives), mismatched related donor (haplo-identical relative), matched unrelated (10/10 identical unrelated donor) and mismatched unrelated (≤ 9/10 HLA-matched unrelated donor). Engraftment definitions were in accordance with the EBMT handbook [23]. Acute and chronic graft-versus-host disease (GvHD) were graded according to the modified Seattle respectively the National Institute of Health criteria [24]. Infections, immune dysregulation, malignancy and specific organ damage were documented with respect to the affected pathogens and/or organs involved.

Immune reconstitution was assessed at different time points after HSCT: at 6 to 12 months, 12 to 18 months and/or ‘at latest follow up’ (range 1–15 years). Immunophenotyping included absolute numbers of CD3 + T cells, CD4 + including CD3 + CD4 + CD45RA + CCR7 + (naïve) T cells, CD8 + T cells, CD56 + (± CD16) NK cells and CD19 + B cells. Immune reconstitution was defined according to age-matched healthy control reference values [25]. Chimerism was performed as per center protocols on whole blood, peripheral blood mononuclear cells (PBMC) or otherwise as specified. Full donor chimerism was defined as ≥ 90% donor cells. Information about ongoing immunosuppressive treatment and/or immunoglobulin substitution, at latest follow up, was available for all patients.

## Results

### Patient Population and HSCT Characteristics

#### Patient Population

A total of 18 patients were included in this study, 16 patients with homozygous or compound heterozygous mutations in either *DNMT3B* (ICF1, *n* = 6), *ZBTB24* (ICF2; *n* = 4), *CDCA7* (ICF3; *n* = 4) or *HELLS* (ICF4; *n* = 2) and two patients without a genetic diagnosis (based on clinical criteria (ICFX; ESID Registry criteria [21]). The median age at onset of symptoms was 0.6 years (range 0.3–14.3 years). Median age at (genetic) diagnosis was 2.0 years (range 0–17.2 years). Three patients (P4, P16, P18) were diagnosed in the first months of life via positive family history. Patient characteristics and pre-transplant disease manifestations are summarized in Tables 1 and 2.

**Table 1:** Patients’ and HSCT (baseline) charcteristics

Characteristics—pre-transplant and transplant procedure	*n* = 18
Sex—*n* (%)	
Male	9 (50)
Female	9 (50)
Genetic subtype—*n* (%)	
ICF1	6 (33)
ICF2	4 (22)
ICF3	4 (22)
ICF4	2 (11)
Clinical diagnosis (genetics unknown; ICFX)^	2 (11)
Age (years) at first symptoms—median (range)	0,6 (0,3—14,3)
Age (years) at diagnosis—median (range)	2,0 (0—17,2)
Main problems/indication for HSCT—*n* (%)	
(Recurrent) infections/(combined) immunodeficiency	15 (83)
Immune dysregulation	4 (22)
Myelodysplasia or malignany	3 (17)
Gastro-intestinal problems/failure to thrive	10 (56)
Pre-emptive	2 (11)
Age (years) at HSCT—median (range)	4.3 (0.5—19)
Period of HSCT—*n* (%)	
≥ 2018	8 (44)
2014—2017	4 (22)
≤ 2014	6 (33)
Donor type—*n* (%)	
MSD or MFD	7 (39)
MMFD	1 (6)
MUD	9 (50)
MMUD	1 (6)
Stem cell source—*n* (%)	
Bone marrow (unmanipulated)	10 (56)
Peripheral blood (unmanipulated)	3 (17)
Peripheral blood (ex vivo manipulated)†	5 (28)
Conditioning regimen—*n* (%)	
MAC	9 (50)
RIC	9 (50)
Serotherapy—*n* (%)	16 (89)
ATG	8 (44)
Alemtuzumab	8 (44)
Graft versus host disease prophylaxis—*n* (%)	
None	1 (6)
CsA or tacrolimus	2 (11)
CsA or tacrolimus + MTX or MMF	14 (78)
CsA + cyclophosphamide	1 (6)
Characteristics—post-transplant	
Overall survival—number (%)	15 (83)
Duration (months) of follow up—mean/median (range)	51/58 (0—185)
Engraftment—*n* (%)*	17 (100)
Time (days) to engraftment^#^—median (range)	17 (10—22)
Acute graft versus host disease—*n* (%)*	
No	11 (65)
Yes	6 (35)
*Grade I-II	*4 (24)*
*Grade III	*2 (12)*
Chronic graft versus host disease—*n* (%)	0 (0)
Post-transplant complications—*n* (%)*	
Infectious	12 (71)
*Viral (adenovirus/CMV/EBV, airway-/gastro-intestinal viruses)	*11 (65)*
*Bacterial	*3 (18)*
*Fungal	*1 (6)*
Immune dysregulation	2 (12)
*Thyroid	*2 (12)*
*Skin	*1 (6)*
Endocrine	2 (12)
Malignant	0 (0)
Chimerism^####^—*n* (%)*	
Full donor chimerism (> 90%)	15 (88)
Mixed chimerism	2 (12)
Cellular immune recovery^##^—*n* (%)	
At t = 1 year (*n* = 13)	10 (77)
At latest follow-up (*n* = 14)	13 (93)
Humoral immune recovery^###^—*n* (%)	13 (87)

^clinical diagnosis based on ESID Registry criteria (Seidel et al. JACIP 2019)

*of evaluable patients (*n* = 17)

†ex vivo manipulation: T cell receptor αβ + B cell depletion

^#^ neutrophil count > 0.5 × 10^9^/L

^##^ CD4 + above age-adjusted tresholds

^###^no Ig substitution at latest follow-up (*n* = 15 evaluable patients)

^####^chimerism at latest follow up

**Table 2:** Clinical features and genetic variants of patients with ICF syndrome (pre-HSCT)

UPN	Age at onset^a^	Age at diagnosis^a^	Sex	Genetic subtype	Mutation	Failure to thrive /diarrhea	Infection			bronchiectasis	Immune dysregulation		Myelodysplasia/ malignancy	Other symptoms	Treatment				Reference
							airways	gastro-intestinal	opportunistic^b^		cytopenia	other/organ			infection	immunology	malignancy/HLH	other	
P1	0.5	0.7	F	ICF1	compound heterozygous c.2426A > G, c.1814 T > C	-/-	+	-	+ (PJP)	-	-	-	-		TMP-SMX	IVIG			Gennery 2007
P2	0.5	0.8	M	ICF1	compound heterozygous c.1957G > A, c.2292G > T	+ / +	+	+ (Astrovirus)	+ (AdV)	-	+ (1-lineage)	-	-		TMP-SMX	IVIG			Kraft 2021
P3	14.3	17.2	F	ICF1	compound heterozygous c.1817 T > C, c.610C > T	?	+	-	+ (Candida)	?	?	-	+ (MDS, transfusion dependency and neutropenia)		?	IVIG			
P4	1.9	0.0	M	ICF1	homozygous c.2397–11G > A	+ / +	-	+ (Campylobacter)	-	-	-	-	-		-	IVIG			Gennery 2007
P5	0.3	0.3	M	ICF1	homozygous c.1807G > A	+/-	+	-	+ (PJP)	-	-	-	-		TMP-SMX	IVIG			Gossling 2017
P6	0.6	12.0	F	ICF1	homozygous c.2118C > A	+ / +	+	-	-	+	-	+ (gut)	-		TMP-SMX, azitromycin	IVIG, mesalazin			
P7	1	11.1	M	ICF2	homozygous c.958C > T	-/-	+	-	+ (CMV, EBV)	+	-	-	-	EBV-driven HLH	TMP-SMX, ciprofloxacin, valgancyclovir, itraconazole	IVIG	Steroids, etoposide, ciclosporine		Harnisch 2016
P8	0.3	0.3	M	ICF2	homozygous c.501dup	+ / +	+	-	+ (PJP)	+	+ (3-lineage)	+ (liver)			?	IVIG		special diet (diarrhea)	
P9	0.3	2.6	F	ICF2	homozygous c.1492_1493del	-/-	+	-	+ (CMV, EBV)	-	-	-	+ (EBV-associated large B cell lymphoma)	sepsis	TMP-SMX, acyclovir, caspofungin, fluconazole	IVIG	RTX, chemotherapy (ANHL1131, group B)		Burk 2020
P10	13	14.3	F	ICF2	compound heterozygous c.958C > T, c.1222 T > G	-/-	-	-	+ (EBV)	-	-	-	+ (DLBCL)		TMP-SMX	IVIG	chemotherapy (B-NHL-BFM-2004)		
P11	0.6	5.6	F	ICF3	homozygous c.1114C > T	+ / +	+	+ (Noro-/astrovirus)	+ (PJP, Candida)	+	-	-	-		TMP-SMX, acyclovir, itraconazole	IVIG			
P12	2	8.1	M	ICF3	homozygous c.1114C > T	+ / +	+	-	-	+	-	-	-		-	IVIG		nasogastric tube feeding	
P13	1	15.6	M	ICF3	homozygous c.1077delC	+ / +	+	+ (Norovirus)	-	+	-	+ (liver, kidney)	-	duodenal lymphoid hyperplasia	-	IVIG, azathioprin		TPN	Staudacher 2023
P14	0.7	1.5	M	ICF3	homozygous c.1077delC	+ / +	+	+ (Norovirus)	+ (CMV)	-	-	’ + (liver)	-	enterovirus encephalitis	valgancyclovir	IVIG			Staudacher 2023
P15	0.5	0.8	F	ICF4	compound heterozygous c.2096A > G, c.370 + 2 T > A	+ / +	+	+ (small round structured viruses)	+ (PJP)	-	-	-	-		TMP-SMX, itraconazole	IVIG			Gennery 2007
P16	0.3	0.0	M	ICF4	compound heterozygous c.2096A > G, c.370 + 2 T > A	-/ +	-	+ (Sapovirus)	-	-	-	-	-		TMP-SMX, acyclovir	IVIG			
P17	0.15	3.3	F	ICFX	-	+ / +	+	+ (Sapovirus)	+ (AdV)	-	+ (2-lineage)	+ (gut, skin)	-	pancreatitis	TMP-SMX, itraconazole	IVIG, steroids, azathioprin, tacrolimus		TPN	
P18	0.3	0.2	F	ICFX	-	+ / +	+	-	-	-	-	-	-		TMP-SMX, fluconazole	IVIG			

^a^age in years

^b^in case AdV is mentioned: prolonged, systemic adenoviremia (as a manifestation of immune deficiency)

Diagnosis based on positive family history in P4, P16 and P18

+  = yes;—= no; ? = unknown

*EBV *Epstein Barr virus, *PJP *Pneumocystis jirovecii pneumonia, *AdV *adenovirus, *CMV *cytomegalovirus, *DLBCL *diffuse large B cell lymphoma, *MDS *myelodysplastic syndrome, *HLH *hemophagocytic lymphohistiocytosis, *TMP-SMX *cotrimoxazole, *IVIG* (intravenous immunoglobulin (substitution), *RTX* (rituximab), *MTX *methotrexate, *TPN *total parenteral nutrition

#### Pre-Transplant Disease Manifestations

Pre-transplant infections were documented in all patients and represented the most common indication for HSCT (in 83% of patients). Viral gastro-intestinal infections, airway infections caused by both viral and opportunistic pathogens and systemic infections with EBV, CMV and/or adenovirus were most common. Two-thirds of patients (12/18) had opportunistic infections (PJP, EBV/CMV/adeno-viremia and/or severe candidiasis) and one-third (6/18) was documented to have pre-transplant bronchiectasis. All patients were on immunoglobulin substitution therapy (and, for patients with data available, serum IgG trough levels were within the normal range for age (Table 3)). Two-thirds of patients (12/18) were on antibacterial prophylaxis, one-third (6/18) received antifungal prophylaxis and 5/18 patients (28%) used antiviral prophylaxis (acyclovir). Two patients (P7 and P14) received valganciclovir to treat primary, persisting CMV infection.

**Table 3:** Immune parameters (pre-HSCT)

UPN	Genetic subtype	Immunoglobulin levels (g/L)			B cell count (cells/µl)	T cell count (cells/µl)			NK cell count (cells/µl)
		IgA	IgM	IgG	CD19 or CD20	CD3	CD4	CD8	CD56 +/- CD16
P1	ICF1	< 0,07 (↓)	0,08 (↓)	0,8 (↓)	725	3802	n/a	n/a	138
P2	ICF1	< 0,07	< 0,07 (↓)	< 0,30^a^ (↓)	1119	6892	3963	2809	124 (↓)
P3	ICF1	0.08	0,39 (↓)	3,63 (↓)	46 (↓)	1963	1262	677	84
P4	ICF1	< 0,01 (↓)	< 0,01 (↓)	4,10^a^	502	2480	1876	837	134 (↓)
P5	ICF1	< 0,05 (↓)	< 0,05 (↓)	0,52^a^ (↓)	1330	n/a	1840	450	208
P6	ICF1	0.27	< 0,3 (↓)	4.86	2534	2599	1899	643	n/a
P7	ICF2	0.46	< 0,3 (↓)	2,8 (↓)	450	3750	2010	1650	180
P8	ICF2	< 0,06 (↓)	< 0,05 (↓)	0,38^a^ (↓)	1130	4640	3200	1380	n/a
P9	ICF2	0.76	0,33 (↓)	5.41	735	2134	1390	713	93
P10^b^	ICF2	0,43 (↓)	0,17 (↓)	1,41 (↓)	33 (↓)	410 (↓)	250 (↓)	150 (↓)	23 (↓)
P11	ICF3	< 0,04 (↓)	< 0,05 (↓)	15,7^c^	916	714 (↓)	339 (↓)	266	230
P12	ICF3	n/a	n/a	n/a	n/a	n/a	n/a	n/a	n/a
P13	ICF3	< 0,1 (↓)	0,05 (↓)	12,96^c^	280	400 (↓)	300 (↓)	70 (↓)	160
P14	ICF3	< 0,01 (↓)	0,09 (↓)	5.7	960	2260	1560	610	180
P15	ICF4	< 0,2 (↓)	< 0,2 (↓)	0,3 (↓)	1320	4092	3300	792	528
P16	ICF4	< 0,3	< 0,22	8,05^a^	240	5137	3750	1298	1977
P17^d^	ICFX	< 0,07 (↓)	< 0,05 (↓)	7,15^c^	20 (↓)	650 (↓)	530	110 (↓)	140
P18	ICFX	0,01 (↓)	0,25 (↓)	1,11 (↓)	122 (↓)	2509	887	887	275

^a^maternal IgG

^b^during treatment for B cell malignancy

^c^with Ig substitution

^d^during immunosuppressive treatment

Reference values for IgG, IgA and IgM as described by Bayram et al. Turk J Med Sci (2019) 49: 497–505. ↓ decreased as compared to age-matched healthy controls

Reference values for peripheral T-, B- and NK-cell counts as described by Schatorje et al. Scand J Immunol, 2012. 75(4): p. 436–44. ↓ decreased as compared to age-matched healthy controls

In addition to infectious diarrhea, four patients (P6, P8, P12 and P18) suffered from chronic non-infectious diarrhea/enteropathy. Overall, diarrhea and/or failure to thrive was documented in 13/18 (72%) patients, with need for nasogastric tube or parenteral feeding in 3/18 patients. Enteropathy was reported in 4/5 evaluable ICF1, 4/4 ICF3 and 2/2 ICF4 patients but in only 1/4 ICF2 patients. Enteropathy with or without failure to thrive was reported the second most common indication for HSCT (in 56% of patients).

Six patients (33%) were reported to suffer from manifestations related to immune dysregulation: 1- to 3-lineage cytopenia (*n* = 3; P2, P8, P17), enteropathy/colitis (*n* = 2; P6 and P17), hepatitis (*n* = 3; P8, P13-14), kidney- (*n* = 1; P13) and/or skin (*n* = 1; P17) disease. Of note, only one (P6) of four patients with non-infectious diarrhea (P6, P8, P12, P18) was categorized as ‘auto-immune enteropathy’, suggesting an underestimation of this disease manifestation in the current cohort. Three of six patients with immune dysregulation were treated with immunosuppressive drugs. In four patients (P6, P8, P13, P17; 22%), immune dysregulation was a major indication for HSCT.

Two patients, both ICF2, presented with a hematological malignancy: EBV-driven B cell lymphoma (P9) and diffuse large B cell lymphoma (P10), while another ICF2 patient (P7) presented with EBV-driven HLH. These three patients were treated with (immuno-)chemotherapy to achieve remission before HSCT. One patient (P3, ICF1) suffered from (transfusion-dependent) myelodysplasia.

#### Pre-Transplant Immunophenotype

Information about the pre-transplant immune parameters is summarized in Table 3. Consistent with existing data about humoral immunity in ICF syndrome, all patients suffered from moderate-severe hypogammaglobulinemia in the presence of normal numbers of circulating total B cells (according to age-adjusted reference values (Table 3)). Few patients (4/17) had decreased B cell counts at first analysis. Except for P18, B cell deficiency was likely secondary to myelodysplasia (P3), treatment of malignancy (P10) or immunosuppressive therapy (P17). In 3/3 patients with data available, switched memory B cells (CD19 + IgD-IgM-CD27-) were very low (≤ 1%).

Total numbers of CD3 + T cells, as well as CD3 + CD4 + and CD3 + CD8 + T cells, were in the normal age-adjusted range for 12/16 (evaluable) patients (Table 2). Three of four patients with reduced T cell counts suffered from either malignancy (*n* = 1, P10) or immune dysregulation (*n* = 2, P13 and P17) and two of these patients (P10 and P17) were treated with chemotherapy or immunosuppressants at the time of analysis. The two patients with T cell lymphopenia without chemo- or immunosuppressive therapy were both ICF3 patients, aged 5.5 (P11) and 15 years (P13). Information about pre-transplant naïve T cell numbers and/or mitogen-induced lymphocyte proliferation was unavailable for almost all patients. Absolute T cell counts did not correlate with occurrence of opportunistic infections and/or hematological malignancy. Due to a limited number of ‘older’ patients (age > 10 years) in this cohort, we were unable to evaluate the natural course of T cell lymphopenia over time.

NK cell counts were in the normal range for most (80%) patients. Two of 15 (evaluable) patients, both ICF1 (P2 and P4), had a mild reduction in NK cell counts. The single patient with significantly reduced NK cell counts was pan-lymphopenic due to chemotherapy for B cell lymphoma (P10).

#### HSCT Characteristics

A total of 18 HSCT procedures were performed in this cohort of 18 patients between 2003 and 2021 in 12 different centers (summarized in Table 4). Median age at HSCT was 4.3 years (range 0.5 – 19 years). Donors were MSD/MFD (*n* = 7), MMFD (*n* = 1), MUD (*n* = 9) and MMUD (*n* = 1). The sources of stem cells for HSCT were bone marrow (BM) or peripheral blood (PB) in *n* = 10 and *n* = 8 cases, respectively. Ex vivo TCRαβ/CD19 depletion of the graft was performed in three cases with PB grafts: one MMUD and two MUD cases. There was an equal distribution between MAC (*n* = 9) and RIC (*n* = 9), with a preference for RIC in patients with pre-transplant organ damage. Except for one MMUD TCRαβ/CD19 depleted HSCT, all patients received graft versus host disease prophylaxis with either a single (*n* = 2) or a combination of immunosuppressive drugs (*n* = 15). In 16 patients (89%), serotherapy was applied, with an equal distribution between ATG (*n* = 8) and alemtuzumab (*n* = 8).

**Table 4:** HSCT and patients’ post-HSCT characteristics

UPN	Genetic subtype	HSCT indication	Age at HSCT^a^	Year of HSCT	donor type/ HLA match	Graft	conditioning regimen MAC/RIC	Conditioning regimen drugs	Serotherapy	GVHD prophylaxis	ANC engraftment, day	aGVHD grade	Survival	Infectious complications	Non-infectious complications	Last chimerism, % donor (time after HSCT)			Last follow up (time after HSCT)
																whole blood	PBMC	other (specified)	
P1	ICF1	recurrent infections	1.4	2005	MUD, 10/10	BM	MAC	bu (16 mg/kg)—cy (200 mg/kg)	ATG	CsA, MTX	21	-	Alive	None	Immune thyroiditis (post-transplant; resolved). Primary ovarian failure	100 (12 y)	n/a	n/a	12 y
P2	ICF1	recurrent infections with failure to thrive	1.0	2018	MSD, 10/10	BM	MAC	flu (150 mg/m2)—mel (140 mg/m2)—thiotepa (10 mg/kg)	alemtuzumab	tacro, MTX	13	-	Alive	Systemic adenovirus (pre-HSCT; resolved)	Complete recovery of (pre-transplant) GE symptoms, failure to thrive and immune dysregulation	44 (stable^b^, 2,5 y)	n/a	75 (CD3 +T cells; 2,5 y)	2,5 y
P3	ICF1	MDS	17.2	2006	MUD, 10/10	BM	RIC	flu (160 mg/m2)—thiotepa (15 mg/kg)	ATG	CsA, MTX	18	-	Died (day + 41)	RSV (respiratory failure, death at day + 41)	None	100 (1 mo)	n/a	n/a	1 mo
P4	ICF1	gastro-intestinal infections with failure to thrive/chronic diarrhea	3.6	2004	MSD, 10/10	BM	MAC	bu (380 mg/m2)—cy (200 mg/kg)	-	CsA, MTX	15	-	Alive	Pneumococcal meningitis resulting in severe hearing loss and pubertas praecox)	Immune thyroiditis (still on Thyrax) and vitiligo (both post-transplant and ((retrospectively) proven) donor derived); recovery from failure to thrive	n/a	100 (10 y)	100 (PMC; 10y)	11 y
P5	ICF1	pre-emptive (recurrent infections and failure to thrive)	0.5	2016	MSD, 10/10	BM	MAC	flu (6 mg/kg)—treo (36 g/m2)—thiotepa (8 mg/kg)	ATG	CsA, MTX	22	-	Alive	RSV, Coronavirus and Influenza infections, Rotavirus GE-itis (all resolved)	None	n/a	> 95 (2.3 y)	n/a	2,3 y
P6	ICF1	recurrent infections, failure to thrive/chronic diarrhea, immune dysregulation	13.0	2019	MFD, 10/10	PB	RIC	flu (150 mg/m2)—treo (42 g/m2)	alemtuzumab	CsA, MMF	18	I (skin)	Alive	None	Complete recovery of (pre-transplant) GE symptoms, failure to thrive and immune dysregulation. Complete recovery of acute GVHD	100 (2 y)	n/a	n/a	2 y
P7	ICF2	recurrent infections and HLH	11.4	2011	MSD, 10/10	BM	MAC	bu (480 mg/m2)—flu (160 mg/m2)	-	CsA, MTX	17	I (skin)	Alive	None	Complete recovery of acute GVHD	n/a	100 (8 y)	100 (PMC; 8y)	8,5 y
P8	ICF2	immune dysregulation	19.0	2016	MSD, 10/10	BM	RIC	flu (120 mg/m2)—cy (3000 mg/m2)	ATG	tacro, MMF	21	-	Alive	Systemic CMV (resolved)	Complete recovery of (pre-transplant) immune dysregulation	> 90 (2,2 y)	n/a	100 (CD3+ T cells; 4 mo)	2,2 y
P9	ICF2	recurrent infections, malignancy	3.0	2018	MUD, 10/10	PB	MAC	flu (5 mg/kg)—mel (140 mg/m2)—thiotepa (200 mg/m2)	alemtuzumab	tacro	10	-	Alive	Systemic EBV, CMV, adenovirus infection (all pre-HSCT, all resolved (after DLI))^c^	Seizures (during MTX, resolved)	100 (1 y)	n/a	98/100 (CD3+ T cells/PMC; 1 y)	1,8 y
P10	ICF2	CID with malignancy	15.4	2018	MMUD, 9/10	PB, TCRab/CD19 depletion	MAC	flu (150 mg/m2)—treo (42 g/m2)—thiotepa (300 mg/m2)	ATG	-	11	-	Died (day + 145)	Systemic adenovirus (multi-organ failure/death at day + 145, despite DLI (twice))^d^, Clostridium	None	> 99 (2 mo)	n/a	> 99 (CD3+ T cells and PMC; 2 mo)	4 mo
P11	ICF3	recurrent infections with bronchiectasis and failure to thrive	5.7	2018	MUD, 10/10	PB	RIC	flu (150 mg/m2)—treo (42 g/m2)	alemtuzumab	CsA, MMF	16	III (gut)	Alive	Systemic adenovirus, Norovirus GE-itis (pre-HSCT; both resolved)	Partial recovery of (pre-transplant) GE symptoms: still on PEG-feeding, but gaining weight. Complete recovery of acute GVHD	100 (1 y)	100 (1 y)	100 (PMC; 1 y)	1 y
P12	ICF3	recurrent infections with bronchiectasis and failure to thrive/chronic diarrhea	8.5	2018	MFD, 10/10	PB	RIC	flu (150 mg/m2)—treo (42 g/m2)	alemtuzumab	CsA, MMF	17	I (skin)	Alive	Pulmonary aspergillosis, systemic HHV6 (both resolved)	Complete recovery of (pre-transplant) GE symptoms: off NG tube feeding. Complete recovery of acute GVHD	100 (9 mo)	100 (9 mo)	95 (CD15+ cells; 9 mo)	3 y
P13	ICF3	gastro-intestinal infection with failure to thrive/chronic diarrhea, immune dysregulation	16.9	2018	MUD, 10/10	PB, TCRab/CD19 depletion	MAC	flu (160 mg/m2)—treo (42 g/m2)—thiotepa (10 mg/kg)	ATG	CsA, MMF	10	-	Alive	Norovirus GE-itis (pre-HSCT; resolved)	Hepatopathy with ascites and capillary leak (resolved; mild fibrosis without portal hypertension). Short stature: still on GH analog. Off TPN and NG tube feeding	100 (3,5 y)	n/a	100 (CD3+ T cells and PMC; 3,5 y)	4,3 y
P14	ICF3	recurrent infections, failure to thrive	2.3	2020	MUD, 10/10	PB, TCRab/CD19 depletion	MAC	flu (5 mg/kg)—treo (42 g/m2)—thiotepa (10 mg/kg)	ATG	CsA, MMF	16	-	Alive	Systemic EBV, CMV infection (pre-HSCT; both resolved after DLI), norovirus (pre-HSCT), clostridium (both resolved)	Complete resolution of (pre-transplant) GE symptoms, failure to thrive and immune dysregulation. Moderate-severe disability after pre-transplant enterovirus encephalitis	85 (3 y^e^)	n/a	90/62 (CD3+ T cells/CD34 + cells; 1,5 y)	3 y
P15	ICF4	gastro-intestinal infections with failure to thrive/chronic diarrhea	2.2	2003	MUD, 10/10	BM, RBC depletion	RIC	flu (150 mg/m2)—mel (140 mg/m2)	alemtuzumab	CsA	19	-	Alive	None	Complete recovery of (pre-transplant) GE symptoms and failure to thrive	100 (14 y^f^)	n/a	n/a	15 y
P16	ICF4	pre-emptive	1.5	2009	MUD, 10/10	BM	RIC	treo (36 g/m2)—cy (200 mg/kg)	alemtuzumab	CsA, MTX	12	III (?)	Alive	None	Complete recovery of (pre-transplant) GE symptoms and acute GVHD	100 (10 y)	100 (2 mo)	100 (PMC; 2 mo)	10 y
P17	ICFX	recurrent infections, cytopenia/immune dysregulation, TPN-dependent enteropathy	5.0	2014	MUD, 10/10	PB	RIC	flu (150 mg/m2)—treo (42 g/m2)	alemtuzumab	CsA, MMF	n/a	n/a	Died (day -1)	Sepsis/conditioning-related toxicity (death at day -1)	n/a	n/a	n/a	n/a	n/a
P18	ICFX	recurrent infections	3.5	2015	MMFD, 5/10	BM	RIC	flu (150 mg/m2)—cy (40 mg/kg)	ATG	CsA, post-Cy	22	II (skin only)	Alive	Systemic CMV, Norovirus GE-itis (both resolved)	Complete recovery of acute GVHD	n/a	98 (2 y)	n/a	3,8 y

^a^age in years

^b^stable mixed chimerism after CD34-selected stem cell boost (dose 6,3 × 10e6/kg) at t = 6 months (pre-boost chimerism: 84% donor (whole blood) and 59% donor (CD3 + T cells))

^c^DLI (cell dose unknown) for persistent EBV- and CMV-viremia, within the first month after HSCT

^d^DLI (twice, CD45RA-depleted CD3 + T cells, cell doses unknown) for persistent EBV- and CMV-viremia, at + 26 and + 71 days after HSCT

^e^DLI, four times in total: three times virus-specific T cells (cell doses unknown) for persistent CMV- en EBV-viremia within the first four months after HSCT; once for mixed chimerism (cell dose unknown; pre-DLI chimerism 80% donor (whole blood))

^f^stable donor chimerism after stem cell boost (cell dose unknown) at t = 6 months (pre-boost chimerism 25% donor (whole blood))

*PMC *polymorphonuclear cells

Age at transplant was remarkable high for patients with pre-HSCT bronchiectasis (mean age 12.4 years) and for patients transplanted for immune dysregulation (mean age 13.5 years) and hematological malignancy/HLH (mean age 13.1 years) as compared to patients with other indications for HSCT (mean age 2.0 years).

#### Engraftment, Chimerism and Post-Transplant Complications

Data about engraftment, chimerism and post-HSCT complications are summarized in Table 4. All evaluable patients (P17 excluded, death before graft infusion) engrafted successfully with a median time to engraftment of 17 days (range 10–22 days). With a median follow-up of 2.2 years (range 0.1–14 years), full donor chimerism (> 90% donor) was reported in all but two patients. Five patients received additional cell therapeutic interventions: P2 received a CD34-selected stem cell boost (at six months after HSCT) for mixed chimerism (pre-boost: 59% (T cells) and 84% (whole blood) donor) resulting in stable mixed chimerism at 2.5 years after HSCT (75% (T cells) and 44% (whole blood) donor); P9 received DLI (within the first month after HSCT) for persistent EBV- and CMV-viremia, leading to full clearance; P10 received DLI (at + 26 and + 71 days after HSCT; CD45RA-depleted T cells) for persistent adenovirus infection, but died at day + 145; P14 received virus-specific T cells for persistent CMV and EBV infection (within the first four months after transplant) and additional DLI for mixed chimerism (80% donor, pre-DLI) at 10 months, resulting in complete viral clearance and stable mixed chimerism (85% donor at 3 years after HSCT); P15 received a stem cell boost for mixed chimerism (25% donor, pre-boost) at 6 months resulting in full donor chimerism at 14 years after HSCT.

Acute graft versus host disease (aGVHD) was documented in 6/17 (35%) evaluable patients with skin-only grade I/II aGVHD in *n* = 4 patients (P6, P7, P12 and P18) and grade III (gut) aGVHD in *n* = 2 patients (P11 and P16, both suffering from pre-HSCT infectious diarrhea). Acute GVHD was successfully treated in all affected patients. There were no cases with chronic GVHD.

Infections were the most common post-HSCT complication: in 11/17 patients (65%) the infections were of viral origin and represented predominantly airway infections, gastro-intestinal infections and/or systemic infections with CMV/EBV/adenovirus. In 5/11 patients (P2, P9, P11, P13, P14), these viral infections originated pre-HSCT. Four patients demonstrated de novo post-HSCT infections of non-viral origin: bacterial (*n* = 3; S. pneumoniae (*n* = 1, P4) and Clostridium (*n* = 2, P10 and P14)) or fungal (*n* = 1, Aspergillus (P12)). All surviving patients recovered from infections without sequelae, except for two patients: pneumococcal meningitis resulted in severe hearing loss and precocious puberty in patient P4 and moderate-severe disability resulted from a pre-HSCT viral encephalitis in patient P14. At > 1 year post-HSCT, anti-microbial prophylaxis was continued in *n* = 4 patients (P6, P8, P11, P12), all with pre-transplant bronchiectasis.

Post-HSCT outcome of pre-existing immune dysregulation was evaluable in 5 survivors (P2, P6, P8, P13, P14). Immune-mediated cytopenia resolved in all evaluable cases (P2, P8) and recovery from hepatitis was documented for 3/3 patients (P8, P13, P14), with residual mild fibrosis without portal hypertension in P13. Data on recovery from pre-HSCT (non-infectious) enteropathy with or without failure to thrive was available for 9/13 survivors: diarrhea resolved in all patients, and 2/2 nasogastric tube and/or parenteral feeding dependent patients (P12 and P13) were off supplemental nutrition at latest follow-up. One patient (P11) was reported to be on PEG-feeding despite clearing Norovirus and gaining weight at 1 year post-HSCT. Two patients developed de novo post-HSCT immune dysregulation: (transient) thyrotoxicosis in P1 and thyroiditis plus vitiligo in P4. Concerning P4: although the HLA-identical sibling donor of this patient appeared healthy at time of stem cell donation, it was (retrospectively) demonstrated that she suffered from antibody-mediated (subclinical) hypothyroidism at time of donation. Therefore, P4 was diagnosed with donor-derived auto-immunity. At latest follow-up, none of the patients were on immunosuppressive drugs.

#### Survival Analysis

**Fig. 1:**
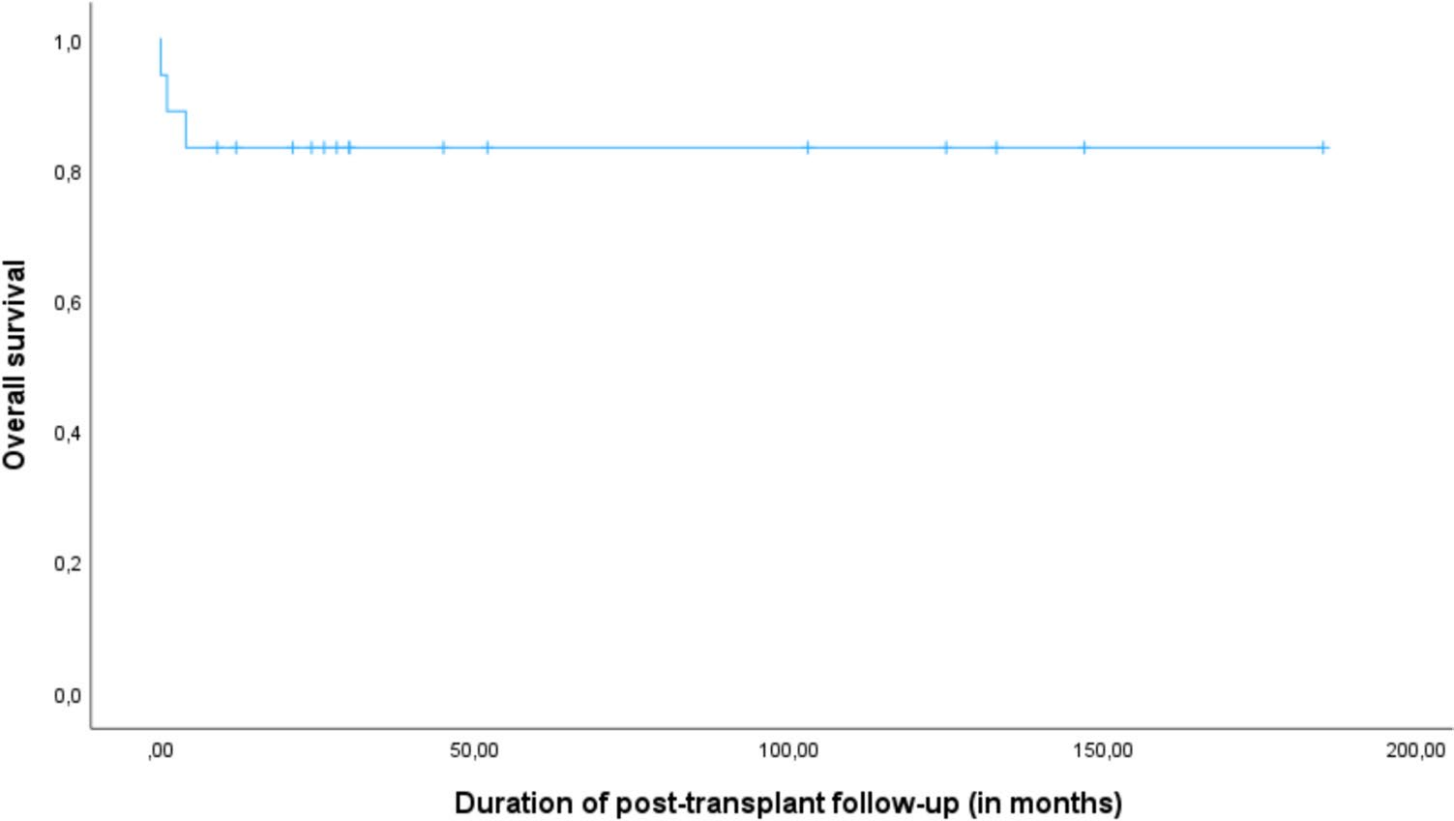
Post-HSCT overall survival

The mean follow-up after transplant was 51 months (range 0–185 months, median 58 months; Table 4). Overall survival was 83%, all deaths occurred within the first five months after HSCT (Fig. 1). Event-free survival (with events defined as death, GvHD > grade II (*n* = 2) and/or graft failure (*n* = 0)) was 72%, with resolution of GvHD in all patients. Three patients died during the HSCT trajectory: P3 (ICF1) died at day 41 after transplant, while being full donor, from respiratory failure most likely due to RS virus infection. P10 (ICF2) died at day + 145 from multi-organ failure after prolonged adenovirus infection, despite early engraftment, full donor chimerism, antiviral therapy and DLI (twice). P17 (ICFX) died one day before graft infusion, due to conditioning related toxicity and sepsis despite having received RIC (Table 4). All three patients who had been diagnosed by positive family history and were transplanted at young age (≤ 3 years), survived.

#### Immune Reconstitution

Cellular immune reconstitution data were collected at 1 year and/or at latest follow-up after HSCT (Table 5). CD3 + T cells numbers were within the age-adjusted reference values at 1 year and at latest follow-up in 92% and 100% of patients respectively (*n* = 13 evaluable patients). A similar favourable pattern was found for CD4 + T cells numbers with 77% and 93%, and for CD8 + T cells counts with 100% and 93%, respectively (*n* = 14 evaluable patients). In 3/8 evaluable patients, naïve CD4 + T cell counts at latest follow-up were within age-adjusted reference values. Median age at HSCT for these three patients was 2.2 years (P14-16), as compared to 8.5 years for patients with naïve CD4 + T cell counts below age-adjusted thresholds (P1-2, P6, P12-13). NK cell reconstitution at 1 year and at latest follow-up showed values within the age-adjusted reference range in 92% of evaluable patients. Finally, B cell numbers were within the age-adjusted reference values in all (*n* = 15) evaluable cases at 1 year and at latest follow-up after HSCT. At latest follow-up, all patients had become immunoglobulin substitution independent, except for two: one patient treated pre-HSCT with high-dose rituximab for hematological malignancy (P9) and one patient with only 9 months post-HSCT follow-up (P12). In both patients, switched memory B cells were still absent (data not shown). Adequate responses to tetanus and pneumococcal (re)vaccination were reported in 8/8 evaluable patients, including a patient with stable mixed chimerism (P2).

**Table 5:** Post-HSCT immune reconstitution

UPN	Genetic subtype	T cell count (at + 1 yr, cells/µl)			NK cell count (at + 1 yr, cells/µl)	B cell count (at + 1 yr, cells/µl)	T cell count (at latest FU, cells/µl)				NK cell count (at latest FU, cells/µl)	B cell count (at latest FU, cells/µl)	Ig dependency (at latest FU)
		CD3	CD4	CD8	CD56 ± CD16	CD19 or CD20	CD3	CD4		CD8	CD56 ± CD16	CD19 or CD20	
							total		naive				
P1	ICF1	996	599	368	135	567	880	608	70 (↓)	198 (↓)	198	297	-
P2	ICF1	2254	1312	853	74 (↓)	494	1641	655	166 (↓)	752	102	468	-
P3	ICF1	n/a	n/a	n/a	n/a	n/a	n/a	n/a	n/a	n/a	n/a	n/a	n/a
P4	ICF1	744 (↓)	372 (↓)	329	129	515	1145	438	n/a	616	249	525	-
P5^a^	ICF1	n/a	n/a	n/a	n/a	n/a	n/a	818	n/a	613	n/a	588	-
P6	ICF1	1910	474	1377	267	1405	1424	523	185 (↓)	834	281	551	-
P7	ICF2	1308	313 (↓)	850	119	382	779	315 (↓)	n/a	401	75 (↓)	217	-
P8	ICF2	1174	313 (↓)	803	454	734	1768	666	n/a	999	378	878	-
P9	ICF2	4367	1339	2998	101	663	3247	1218	n/a	1996	n/a	n/a	+ ^b^
P10	ICF2	n/a	n/a	n/a	n/a	n/a	n/a	n/a	n/a	n/a	n/a	n/a	n/a
P11	ICF3	2016	1017	901	115	540	n/a	n/a	n/a	n/a	n/a	n/a	-
P12^c^	ICF3	n/a	n/a	n/a	n/a	n/a	830	330	83 (↓)	468	220	316	+
P13	ICF3	930	450	410	130	650	760	420	53 (↓)	270	110	480	-
P14	ICF3	1539	900	560	240	600	3000	1550	878	1300	150	390	-
P15	ICF4	2269	1447	730	187	326	1868	1160	318	547	543	354	-
P16	ICF4	2398	1459	741	448	1046	1753	934	278	630	453	616	-
P17	ICFX	n/a	n/a	n/a	n/a	n/a	n/a	n/a	n/a	n/a	n/a	n/a	n/a
P18	ICFX	2155	1367	890	209	186	1311	808	n/a	496	153	387	-

At latest follow up: all patients off immunosuppressive drugs

^a^latest data: (only) 6 months after transplant

^b^after high-dose Rituximab for lymphoma treatment

^c^latest data: (only) 9 months after transplant

Vaccination responses: normal for all patients evaluated (P1, 2, 4, 5, 7, 13, 15, 16)

Reference values for peripheral T-, B- and NK-cell counts as described by Schatorje et al. Scand J Immunol, 2012. 75(4): p. 436–44. ↓ decreased as compared to age-matched healthy controls

## Discussion

While detailed clinical, genetic and immunological characteristics of patients with ICF syndrome have been reported in several studies [1, 2, 6–12], the published experience on HSCT in these patients has been limited to selected cases [2, 8, 11, 15–20]. In this joint study, we report the largest international cohort of transplanted ICF patients and provide comprehensive data on their clinical, genetic, immunological and favourable HSCT outcome characteristics.

Our study cohort encompasses the full spectrum of the four known genetic subtypes of ICF syndrome, as well as genetically undetermined patients. In line with the defined ICF characteristics, all patients had moderate to severe hypogammaglobulinemia in the presence of normal B cell numbers and were immunoglobulin substitution dependent. Infections, often presenting already in infancy or early childhood, represented the most common indication for HSCT. While this was expected based on the typical and profound humoral immune deficiency in ICF syndrome, remarkably two-thirds of the patients had encountered opportunistic infections indicative for a broader adaptive immune deficiency, likely involving T cell deficiency as previously suggested [1, 2, 6–12]. Only a minority of patients in this cohort had quantitative T cell deficiency. Functional T cell studies were not available, due to the retrospective nature of the study.

Overall survival at 1 year after transplantation as well as at latest follow-up (mean 4.5 years after HSCT) in this ICF syndrome patient cohort was 83%. Engraftment was uncomplicated in all evaluable cases and full donor chimerism was reported in all but two patients. Adequate cellular immune reconstitution, if defined as CD3 + and CD4 + T cell counts above age-adjusted reference values, was reported in the majority of patients at + 1 year and at latest follow-up after HSCT. Adequate naïve CD4 + T cell counts at latest follow-up were reported in 3/8 patients with data available and predominantly in younger patients (median age 2.2 years as compared to median age 8.5 years for patients with reduced naïve CD4 + T cell counts). A possible role for thymic damage due to pre-transplant immune dysregulation, infections and/or (immune-/chemo)therapies in reduced immunological reconstitution can be conceptualized in older patients [26, 27]. With regard to humoral immune recovery, all but two (13/15) patients were off immunoglobulin substitution therapy and adequate responses to post-transplant tetanus and pneumococcal vaccination were reported in 8/8 patients with data available.

Following HSCT and concomitant reconstitution of donor immunity, both chronic persistent and opportunistic infections were successfully cleared/eradicated. Similar to the infectious disease manifestations, non-infectious enteropathy responded very well to the HSCT procedure and stable remission was achieved in all evaluable patients, including 2/2 patients with pre-transplant nasogastric tube and/or parenteral feeding, indicating that impaired immune function rather than a non-immunological (gut-intrinsic) factor was causative for the enteropathy and associated failure to thrive. Immune dysregulation represented an important indication to proceed towards HSCT, especially in older ICF patients. In all patients with pre-existing immune dysregulation, stable remission was achieved following HSCT without the need for continuation of immune suppression. None of the evaluable patients with initial myelodysplasia or hematological malignancy/HLH presented disease recurrence after HSCT (only 2/4 patients survived the procedure). Together, this emphasizes that HSCT is effective to cure the main disease manifestations and indications for transplant in patients with ICF syndrome.

The unfavourable impact of pre-HSCT infections and other co-morbidities on HSCT outcome regarding survival, transplant complications and quality of life has been demonstrated for a number of inborn errors of immunity at paediatric, adolescent and adult age [28–30]. Despite the considerable infectious and non-infectious comorbidity in this ICF cohort, HSCT outcome is relatively favourable. Particularly, the rather low incidence of aGvHD and the absence of cGvHD are remarkable. This may be related to the use of RIC regimens in vulnerable patients and the predominance of HLA matched donors. Importantly, two patients with mixed chimerism > 2.5 years after HSCT (44 – 85% donor chimerism) did demonstrate a favourable outcome with adequate immune reconstitution and resolution of (pre-/post-transplant) infections, immune dysregulation, enteropathy and failure to thrive, which further supports the use of reduced-intensity conditioning regimens to reduce treatment-related toxicity and longer-term complications in the most vulnerable patients.

Age at HSCT has been reported as a prognostic factor, although this is probably at least partly linked to co-morbidity. Within our cohort, first symptoms of ICF syndrome often manifested during infancy or early childhood, but genetic diagnosis was frequently delayed with a range of up to 14 years. This delay may be related to the rarity of the disease (resulting in delayed recognition), improved access to next-generation sequencing in the past decade and the rather recent identification of additional genetic subtypes [3] that identified and diagnosed several previously undiagnosed (or ICFX) patients as ICF3 or ICF4 patients. Few patients were diagnosed in the first months of life based on positive family history. One of these patients underwent pre-emptive HSCT at age 1 year, in good clinical condition and with a rather uneventful course and excellent outcome at 10 years after HSCT. The two other patients were transplanted at age 3 years for recurrent infections ± failure to thrive, despite antimicrobial prophylaxis and immunoglobulin substitution therapy. Although both patients survived, one patient experienced long-term sequelae after post-transplant pneumococcal meningitis and (proven donor-derived) auto-immunity.

Although the limited sample size of this study did not allow an age-related outcome analysis, our patient cohort did demonstrate an increased prevalence of organ damage including bronchiectasis and immune-mediated colitis/hepatitis/nephritis as well as haematological malignancies in older patients and age at transplant was remarkably higher in patients transplanted for immune dysregulation and/or haematological malignancy as compared with patients transplanted for other indications. Moreover, two of three patients that died were transplanted at relatively old age (age > 15 years, for myelodysplasia and after treatment for diffuse large B cell lymphoma). The third patient was transplanted at younger age (5 years), but suffered from severe pre-transplant immune dysregulation including TPN-dependent colitis and died from (reduced intensity) conditioning-related toxicity.

Reduced numbers of (naïve) T cells and immune-mediated phenomena, with an increasing prevalence over time, have been reported for patients with ICF syndrome and are considered part of the combined immunodeficiency phenotype of this disease. The progressive T lymphopenia and/or defective T lymphocyte proliferation is reported to be due to either poor T cell neogenesis and/or accelerated apoptosis of T cells [2, 6, 7, 9, 10, 26]. In addition, a recent publication demonstrated reduced regulatory T cell counts in patients with ICF syndrome [12]. A correlation between occurrence of immune dysregulation and reduced T cell counts and/or T cell proliferation has been described in specific patients. Moreover, the lack of detectable auto-antibodies, detection of T cell infiltrates in affected tissues and the beneficial effects of infection prophylaxis and/or immunosuppressive drugs on patients’ clinical condition suggest a major role for T cells in ICF syndrome-related immune dysregulation [1, 2, 6, 8–11, 15]. This supports the curative potential of HSCT to overcome the immune disorder in ICF syndrome.

The combined immunodeficiency phenotype of ICF syndrome is reflected by the infectious and immune-mediated disease burden in our cohort of ICF syndrome patients: both recurrent airway- and gastro-intestinal infections, predominantly attributed to humoral immune deficiency, and opportunistic infections including *Pneumocystis jirovecii* pneumonia (PJP) and systemic viral infections (CMV, EBV, adenovirus) were reported. Most patients with opportunistic infections demonstrated T cell counts within the normal reference ranges for age. A similar pattern was observed for patients with immune dysregulation. Moreover, despite high sensitivity of TREC screening in patients with severe combined immunodeficiencies and absolute T lymphopenia, patients with ICF syndrome are most commonly born with normal counts of naïve T cells and will present with TREC levels above cut-off values at birth [11]. Although the exact pathophysiology, including the contribution of the different genetic defects, underlying T cell dysfunction in ICF syndrome needs to be further clarified, current data are strongly suggestive of a dominant role for T cell dysfunction rather than T cell lymphopenia. Therefore, early recognition of ICF syndrome as a disease causing combined immunodeficiency by clinicians seems crucial for early diagnosis and timely initiation of therapies including (pre-emptive) HSCT.

Reduced NK cell numbers and decreased NK cell function have been described in patients with ICF syndrome with an apparent overrepresentation of ICF2 patients, in particular those with EBV-driven lymphoma or HLH [8, 9, 12, 16, 19, 20]. Interestingly, within our patient cohort, all (*n* = 3) patients with EBV-driven lymphoma or HLH were indeed ICF2 patients. Previous reports have highlighted the importance of cellular immunity, including NK cell function, in anti-EBV immunity (as reviewed by [31]). Based on these findings and reports, a role for *ZBTB24* in NK cell maturation and function has been suggested, but is as yet poorly understood and requires further investigations [6, 19].

Our results support the concept that poor prognosis of ICF syndrome patients is caused by a combined immunodeficiency, with progressive immune dysfunction over time resulting in cumulative morbidity including bronchiectasis, immune-mediated organ damage and/or (haematological) malignancies. Overall survival rates of 60–84% are reported in several (older) cohorts of predominantly non-HSCT-treated ICF patients [1, 9, 14]. Our data demonstrate that HSCT with either myeloablative or reduced-intensity conditioning has the potential to fully correct this combined immunodeficiency without significant demonstrable toxicity and results in adequate immune reconstitution with resolution of pre-transplantation infections, immune dysregulation including non-infectious enteropathy and malignancy, even in the presence of mixed donor chimerism. These encouraging results argue in favour of timely consideration of HSCT in patients with ICF syndrome.

## Data Availability

No datasets were generated or analysed during the current study.
